# Early life Triclosan exposure and child adiposity at 8 Years of age: a prospective cohort study

**DOI:** 10.1186/s12940-018-0366-1

**Published:** 2018-03-05

**Authors:** Geetika Kalloo, Antonia M. Calafat, Aimin Chen, Kimberly Yolton, Bruce P. Lanphear, Joseph M. Braun

**Affiliations:** 1 Department of Epidemiology, Brown University, Providence, RI USA; 2 Centers for Disease Control and Prevention, National Center for Environmental Health, Atlanta, GA USA; 3 Department of Environmental Health, University of Cincinnati, Cincinnati, OH USA; 4 Department of Pediatrics, Cincinnati Children’s Hospital Medical Center, University of Cincinnati College of Medicine, Cincinnati, OH USA; 5 Child and Family Research Institute, BC Children’s and Women’s Hospital, Vancouver, BC Canada; 6 Canada Faculty of Health Sciences, Simon Fraser University, Burnaby, BC Canada; 7 Brown University School of Public Health, Box G-S121-3, Providence, RI 02912 USA

**Keywords:** Environmental exposures, Triclosan, Adiposity, Endocrine disruptors, Prenatal exposure

## Abstract

**Background:**

Triclosan is an antimicrobial agent that may affect the gut microbiome and endocrine system to influence adiposity. However, little data from prospective studies examining prenatal and childhood exposures exist. We investigated the relationship between multiple, prospective early life measure of triclosan exposure and child adiposity.

**Methods:**

In a prospective cohort of 220 mother-child pairs from Cincinnati, OH (enrolled 2003–2006), we quantified triclosan in urine samples collected twice during pregnancy, annually from 1 to 5 years of age, and once at 8 years. We assessed child adiposity at age 8 years using body mass index (BMI), waist circumference, and bioelectric impedance. We estimated covariate-adjusted associations of child adiposity with a 10-fold increase in average prenatal, average early childhood (average of 1–5 years), and 8-year triclosan concentrations.

**Results:**

Among all children, there was no association between triclosan and child adiposity. While urinary triclosan concentrations at all three time periods were weakly, imprecisely, and inversely associated with all three measures of adiposity among girls, these associations did not differ significantly from those in boys (sex x triclosan *p*-values> 0.35). Among girls, the strongest associations were generally observed for prenatal triclosan when we adjusted for all three triclosan concentrations and covariates in the same model; BMI z-score (β: -0.13; 95% CI: -0.42, 0.15), waist circumference (β: − 1.7 cm; 95% CI: -4.2, 0.7), and percent body fat (β :-0.6; 95% CI: -2.7, 1.3). In contrast, the associations between triclosan concentrations and adiposity measures were inconsistent among boys.

**Conclusion:**

We did not observe evidence of an association of repeated urinary triclosan concentrations during pregnancy and childhood with measures of child adiposity at age 8 years in this cohort.

**Electronic supplementary material:**

The online version of this article (10.1186/s12940-018-0366-1) contains supplementary material, which is available to authorized users.

## Background

Childhood obesity is a major public health problem. Over the last 30 years the prevalence of childhood obesity has doubled in the United States; in 2012, over one-third of children and adolescents were either overweight or obese [1]. Furthermore, obesity is an escalating issue across the globe; the prevalence of childhood obesity is increasing in India, China, and Brazil [2]. Increased adiposity can elevate the risk of cardiovascular disease, type 2 diabetes, bone and joint problems, and premature mortality [3]. While excess food consumption, inadequate physical activity, and genetics are risk factors for childhood obesity, recent studies suggest that environmental chemicals may act as obesogens to increase the risk of obesity [4, 5].

Triclosan is a widely used antimicrobial chemical found in some household and personal care products, including toothpaste, mouthwash, soaps, deodorants, textiles, toys, medical devices, and kitchenware [6, 7]. Approximately 75% of all adults and children over the age of 6 and over 85% of pregnant women in the United States have detectable concentrations of triclosan in their urine [8, 9]. Triclosan may impact obesity risk either by changing the composition of the gut microflora or endocrine disruption. Studies in fish and rodents report that triclosan exposure significantly affected gut microbiome composition; [10, 11] but a small crossover study in adults did not [12]. Disruption of the gut microbiome and gut dysbiosis may be associated with increased risk of childhood obesity [10]. Additionally, triclosan may affect thyroid hormone homeostasis [7, 13]. Disruption of maternal thyroid hormones during pregnancy may be associated with body mass index (BMI) among children [14]. Furthermore, disruption of endogenous thyroid hormones has been associated with excess adiposity in adults [15, 16].

Previous studies examining the association between triclosan exposure and adiposity or obesity risk have produced inconclusive results. Among five cross-sectional studies, two reported that triclosan was associated with decreased adiposity, another reported that triclosan concentrations were associated with increased BMI, and two others reported no associations [17–21]. Among two prospective cohort studies, neither found associations between prenatal urinary triclosan concentrations and childhood adiposity or weight [22, 23].

Prior studies relied on the triclosan concentration in one maternal urine sample late in pregnancy or one sample obtained at the time of the adiposity measurement, and thus did not comprehensively estimate triclosan exposure. In addition, we are not aware of any prospective studies examining the association between early childhood triclosan exposure and childhood obesity. To address these gaps, we examined the relationship between prenatal, early childhood, and concurrent urinary triclosan concentrations with measures of adiposity at age 8 years. Because triclosan may disrupt the gut microbiome or endocrine system of children, we expected to observe higher levels of adiposity among those children with high maternal or early childhood urinary triclosan concentrations.

## Methods

### Study participants

The Health Outcomes and Measures of the Environment (HOME) Study recruited women from Cincinnati area prenatal clinics from March 2003 through January 2006. Briefly, women enrolled were 18 years or older, approximately 16 weeks of gestation, living in a home built before 1978, not on medications for thyroid disorders or seizures, planning to continue prenatal care and deliver at the collaborating clinics and hospitals, planning to live in the Cincinnati area for the next year, fluent in English, and had no diagnosis of diabetes, bipolar disorder, schizophrenia, HIV infection, or cancer that resulted in radiation treatment or chemotherapy. Of the 1263 eligible women, 468 women enrolled in our study (37%), 67 dropped out before delivery, and there were 3 stillbirths and 9 sets of twins. The remaining 389 mother-child pairs delivered a liveborn singleton infant. Details regarding eligibility criteria, participant recruitment, and follow-up have been described previously [24]. For the purpose of this analysis, we included participants who had completed the 8-year follow up visit where body composition was measured, complete covariate data, and at least one urinary triclosan measurement during pregnancy, between 1 to 5 years of age, or at 8 years of age.

The institutional review boards (IRB) of Cincinnati Children’s Hospital Medical Center (CCHMC), the hospitals at which the children were delivered, and the Centers for Disease Control and Prevention (CDC) approved this study protocol. The IRB at Brown University relinquished authority to the CCHMC IRB through an Interagency Agreement. Written informed consent was provided by all women for themselves and their children.

### Maternal and child Triclosan exposure assessment

Mothers provided two urine samples in polypropylene cups at approximately 16 and 26 weeks of gestation. Children provided spot urine samples at clinic or home visits annually from 1 to 5 years of age and at age 8 years. For children who were not toilet trained, a surgical insert was placed into a clean diaper at the beginning of the study visit and checked at the end of study visit for urine. For children who were being toilet trained, we lined a training toilet with inserts and collected urine from the inserts. For toilet-trained children, urine samples were collected directly into polypropylene cups with the help of the child’s caregiver. All samples or inserts were stored at − 20 °C and shipped on dry ice to the CDC for analysis.

Total (conjugated + free) triclosan concentrations were measured in urine using online solid phase extraction coupled with high performance chromatography isotope dilution-tandem mass spectrometry using previously described analytic methods [25]. For prenatal and 1 to 5 year urine samples, the limit of detection (LOD) was 2.3 ng/ml; the LOD for samples collected at age 8 years was 1 ng/ml. The change in the LOD was due to the development of a more sensitive method of quantifying triclosan in the samples collected at 8 years of age. All values below the LOD were given a value of LOD/√2 in statistical analyses [26]. To control for individual variation in urine dilution, triclosan concentrations were divided by urinary creatinine concentrations and multiplied by 100 (μg triclosan/g creatinine). To reduce the influence of extreme triclosan concentrations, we log_10_-transformed creatinine-standardized triclosan concentrations.

We categorized urinary triclosan concentrations during three time periods of development: prenatal, early childhood, and concurrent. We averaged maternal creatinine-standardized urinary triclosan concentrations at 16 and 26 weeks of gestation to create an estimate of prenatal triclosan exposure. The early childhood measure was an average of available creatinine-standardized child urinary triclosan concentrations from 1 to 5 years of age (see Additional file 1: Table S1 for the number of children with 1–5 samples). Concurrent triclosan exposure was assessed using creatinine-standardized urinary triclosan concentrations from the 8-year visit.

### Child adiposity assessment

Weight, height, waist circumference, and body fat percentage at the 8-year visit were each measured in triplicate, and the three values of each measure were averaged. We measured child weight with the child dressed in light clothes using a digital scale. We used an Ayrton Stadiometer Model S100 to measure standing height with the child standing straight with their heels against the wall, without any shoes or head coverings. We calculated BMI and then converted it to sex- and age-specific z-scores using U.S. reference values available through the National Center for Health Statistics as described previously [27]. We measured waist circumference by placing a plastic measuring tape around the horizontal plane defined by the child’s left and right iliac crests. We estimated body fat percent by bioelectric impedance using a Tanita children’s body fat monitor. Tanita bioelectric impedance analysis has been shown to be highly correlated to measurements determined through Dual-energy X-ray absorptiometry, Pearson’s *r* > 0.90 [28]. All research staff were blinded to mothers’ and children’s urinary triclosan concentrations when collecting anthropometric measures.

### Covariates

Research staff abstracted medical records and surveyed participants using computer-assisted questionnaires to ascertain maternal and child sociodemographic, perinatal, and behavioral/lifestyle factors at baseline and 8 years of age. Sociodemographic variables included maternal race, age, education, marital status, and household income. Perinatal factors included maternal age at delivery, maternal BMI, prenatal vitamin use, delivery method, breastfeeding, parity, gestational diabetes, and hypertensive disorders. We used maternal serum concentrations of cotinine, a metabolite of nicotine, to assess prenatal tobacco smoke exposure [29]. Lifestyle factors included parent report of children’s hours watching television, hours playing outside, and frequency of fruit, vegetable, and fish consumption at age 8 years; these variables were used to create diet and physical activity variables. Separate directed acyclic graphs (DAG) for prenatal and childhood triclosan exposure were used to select the most parsimonious set of confounding factors including maternal race, education, age at delivery, prenatal vitamin use, marital status, household income, delivery method, maternal BMI, and serum cotinine concentrations (Additional file 1: Figs. S1, S2). We also adjusted for the children’s age when examining waist circumference and body fat percentage.

### Statistical analyses

We began by calculating the geometric mean and median urinary triclosan concentrations for each visit and mean BMI z-scores at 8 years of age for categories of the covariates. We calculated intraclass correlation coefficients (ICCs) between repeated prenatal or early childhood log_10_-transformed creatinine-standardized urinary triclosan concentrations to quantify the reproducibility of these measures and determine if averaging concentrations within a given period would potentially reduce exposure misclassification [30]. Then, we calculated Pearson correlation coefficients between the prenatal, early childhood, and 8-year urinary triclosan concentrations to determine if urinary triclosan concentrations at different periods of development could confound one another because of exposure sources in a child’s household that are maintained over time. We assessed linearity between urinary triclosan and adiposity with restricted cubic polynomial splines [31]. All non-linearity *p*-values were ≥ 0.12, so we fit our models with continuous, log_10_-transformed creatinine-standardized urinary triclosan concentrations as the exposure.

We then examined whether prenatal, early childhood, or concurrent urinary triclosan concentrations were associated with differences in BMI z-score, waist circumference, and percent body fat at age 8 years using multivariable linear regression.

To mitigate concerns that urinary triclosan concentration during one period may be confounding triclosan-adiposity associations at another period, we also adjusted for prenatal, early childhood, and concurrent urinary triclosan concentrations simultaneously in the same covariate-adjusted model. Because prior studies have observed sex-specific associations between triclosan and health outcomes, we examined whether the association between urinary triclosan concentrations and child adiposity was stratified by child sex, using a multivariable model with all covariates [32]. Effect measure modification (EMM) was assessed using a two sample z-test to compare the effect estimates for boys and girls [33]. Significant modification was identified by EMM *p*-values ≤ 0.2 [34].

### Secondary analyses

In secondary analyses, we excluded infants born small for gestational age (birth weight z-score ≤ 10th percentile for gestational age) since low birth weight infants have been shown to have different adiposity distribution and rates of growth [35, 36]. We also conducted analyses where we excluded children born to women with gestational hypertensive disorders and diabetes because these disorders may be associated with child adiposity or triclosan excretion during pregnancy. We also included breastfeeding and diet and physical activity as covariates in the model as they have been associated with child adiposity. In order to assess whether prenatal exposure to other potentially obesogenic chemicals confounded the association between triclosan and adiposity, we adjusted for prenatal urinary concentrations of bisphenol A (BPA), the sum of four di(2-ethylhexyl) phthalate (∑DEHP) metabolites, monobenzyl phthalate (MBzP), monoethyl phthalate (MEP), mono(2-carboxypropyl) phthalate (MCPP), mono-n-butyl phthalate (MnBP), mono-iso-butyl phthalate (MiBP) and of serum perfluorooctanoic acid (PFOA) and brominated diphenyl ether-47 (BDE-47) in separate models [37–39].

Finally, to determine if different statistical methods to control for urine dilution impacted our results, we used a number of previously described dilution adjustment methods [40]. These included: 1) no creatinine-standardization or adjustment, 2) adjusting for creatinine as a covariate in the analysis with unstandardized urinary triclosan concentrations, in which creatinine concentrations were averaged for prenatal and early childhood periods, 3) creatinine-standardization of urinary triclosan concentrations plus creatinine covariate adjustment.

SAS version 9.4 (SAS Institute, Inc. Cary, NC) was used for all statistical analysis.

## Results

Of the 389 singleton infants born to women who enrolled in the HOME Study, 220 (57%), 212 (54%), and 218 (56%) had prenatal, early childhood, and 8-year exposure data, respectively, as well as complete adiposity and covariate data. The mothers in this analysis were predominately non-Hispanic white (60%), college educated (46%), multiparous (55%), and married (61%) (Table 1). Comparing baseline characteristics, women and their children who returned at the 8-year clinic visit were similar to the original cohort [24].

**Table 1:** Geometric mean urinary triclosan concentrations and mean child BMI z-score at by covariates^a,b^

Covariate	*N* (%)	Prenatal Geometric Mean Triclosan (ng/mL)	Early Childhood Geometric Mean Triclosan (ng/mL)	8-year Geometric Mean Triclosan (ng/mL)	8-year child BMI z-score Mean (SD)
All	220	17	11	12	0.36 (1.00)
Maternal Age					
18–25 years	59 (27)	18	8	9	0.41 (1.08)
> 25–35 years	130 (59)	18	13	14	0.38 (0.98)
> 35 years	31 (14)	16	7	13	0.21 (0.94)
Maternal Race					
White	132 (60)	16	11	14	0.21 (0.95)
Black	77 (35)	20	10	10	0.65 (0.99)
Other	11 (5)	17	10	7	0.24 (1.37)
Maternal Education					
Bachelor’s/Grad/Prof	101 (46)	17	12	15	0.34 (0.92)
Tech school/Some College	62 (28)	20	12	13	0.08 (1.09)
High School	33 (15)	14	6	9	0.71 (0.90)
< High School	24 (11)	16	9	7	0.75 (1.00)
Marital Status					
Married	135 (61)	18	12	14	0.27 (0.97)
Not married (Living Alone)	26 (12)	17	7	17	0.27 (0.89)
Not married (Living w/ Someone)	59 (27)	16	10	8	0.63 (1.08)
Household Income					
> $80 K	56 (25)	19	13	15	0.27 (0.89)
$40-80 K	71 (32)	17	11	14	0.26 (0.94)
$20-40 K	34 (15)	19	9	16	0.39 (1.09)
< $20 K	59 (27)	16	9	8	0.56 (1.11)
Parity					
Nulliparous	100 (45)	17	11	12	0.28 (1.03)
1 to 2	66 (30)	19	10	12	0.40 (0.98)
3+	54 (25)	18	11	14	0.47 (0.99)
Employment Status					
Unemployed	40 (18)	15	11	10	0.51 (1.08)
Employed	180 (82)	18	11	13	0.33 (0.98)
Delivery Method					
Vaginal	162 (74)	17	10	12	0.33 (0.99)
Cesarean	58 (26)	21	12	14	0.47 (1.05)
Maternal BMI (kg/m^2^)					
< 25	90 (41)	18	11	13	0.01 (0.97)
25–30	73 (33)	20	12	15	0.37 (0.84)
> 30	57 (26)	15	9	9	0.92 (1.00)
Child Sex					
Female	121 (55)	16	11	13	0.43 (1.01)
Male	99 (45)	19	11	11	0.28 (1.00)
Prenatal Vitamin					
Rarely/Never	31 (14)	12	9	7	0.47 (1.20)
Weekly/Daily	189 (86)	18	11	13	0.35 (1.01)
Prenatal Tobacco Smoke Exposure ^b^					
Unexposed	69 (31)	17	10	12	0.21 (0.94)
Second Hand Smoke	124 (56)	19	13	14	0.42 (1.02)
Active	27 (12)	17	8	9	0.49 (1.06)

^a^Prenatal triclosan concentrations derived from the mean of the 16-week and 26-week urinary triclosan concentrations (*N* = 220). Early childhood triclosan concentrations derived from the mean from 1 to 5 year annual urinary triclosan concentrations (*N* = 212). *N* = 218 for 8-year urinary triclosan concentrations

^b^Unexposed: < 0.015 ng/ml serum cotinine was classified as unexposed, Secondhand Smoke: ≤ 3.0 ng/ml serum cotinine, Active Smoking: > 3.0 ng/ml serum cotinine

**Fig. 1:**
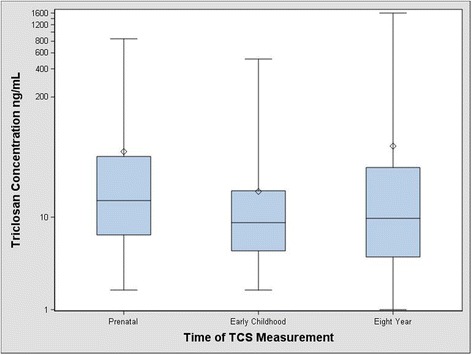
Urinary triclosan concentrations during three developmental periods among women and children from the HOME Study^a,b^. Whiskers represent the minimum and maximum, and the box edges represent the 25th and 75th percentiles, the line in the box is the median and the diamond is the arithmetic mean. The median prenatal triclosan concentrations are higher than triclosan concentrations during either early childhood or 8 years of age. ^a^Prenatal triclosan concentrations derived from the mean of the 16-week and 26-week urinary triclosan concentrations. Early childhood triclosan concentrations derived from the mean of urinary triclosan concentrations taken annually from 1 to 5 years of age and eight year triclosan concentrations were taken concurrently at the time of adiposity measurement. All urinary triclosan concentrations are creatinine standardized. ^b^The minimum for all urinary triclosan concentrations was set to LOD/sqrt(2)

Unstandardized urinary triclosan concentrations among pregnant mothers were higher (Median: 15 ng/ml, Range: < 2.3–853 ng/ml) than their children’s triclosan concentrations during either early childhood (Median 9.0 ng/ml, Range: < 2.3–515 ng/ml) or at age 8 years (Median: 10 ng/ml, Range: < 1.0–1610 ng/ml) (Fig. 1). Prenatal urinary triclosan concentrations were moderately correlated with early childhood triclosan concentrations (Pearson’s *r* = 0.3, *p* <  0.0001) and weakly correlated with 8-year triclosan concentrations (Pearson’s *r* = 0.2, *p* = 0.003). Early childhood triclosan concentrations were moderately correlated with 8-year triclosan concentrations (Pearson’s *r* = 0.4, *p* <  0.0001). The creatinine-standardized urinary triclosan at 16 and 26 weeks of gestation (ICC = 0.51) and at ages 1 to 5 years (ICC = 0.29) had good and poor reproducibility, respectively. Geometric mean unstandardized urinary triclosan concentrations during pregnancy, early childhood and at age 8 years were generally higher among those participants with higher socioeconomic status (e.g., higher maternal education) compared to those with lower socioeconomic status (Table 1).

The mean BMI z-score among children at the 8-year clinic visit was 0.36 standard deviation scores (SD = 1.01); 26% of children in our study were either overweight or obese, characterized as being at or above the 85th percentile of BMI z-score. Children’s BMI z-scores were higher among children delivered by cesarean section and whose mothers were non-Hispanic black, < 25 years old at delivery, had a high school education or less, and made less than $20,000 a year (Table 1). Similar patterns were observed for waist circumference and body fat percentage (results not shown).

After covariate adjustment, we observed no association of prenatal, early childhood, and 8-year urinary triclosan concentrations with children’s BMI z-score, waist circumference, and body fat percentage (Table 2). While the associations between urinary triclosan concentrations and childhood adiposity were not modified by child sex (EMM *p*-values = 0.37 to 0.93) (Table 2), among girls, we consistently observed inverse, albeit weak and imprecise, associations of prenatal, early childhood, and 8-year urinary triclosan concentrations with all three measures of body composition (Table 2). Additionally, the associations of prenatal urinary triclosan concentrations with childhood BMI and waist circumference among girls became slightly stronger after adjustment for triclosan concentrations measured at other times (Table 3).

**Table 2:** Adjusted difference in adiposity measures at age 8 years per 10-fold-increase in urinary triclosan concentrations among HOME Study children^a,b^

Adiposity Outcome and Triclosan Measurement Timing	All, Adjusted Difference (95% CI)	Boys, Adjusted Difference (95% CI)	Girls, Adjusted Difference (95% CI)	EMM *p*-value^c^
BMI z-score				
Prenatal	−0.03 (− 0.24, 0.19)	0.07 (− 0.24, 0.38)	−0.12 (− 0.40, 0.16)	0.37
Average 1–5 years	0.05 (− 0.27, 0.37)	0.04 (− 0.50, 0.59)	− 0.12 (− 0.49, 0.25)	0.50
8 Years	− 0.05 (− 0.23, 0.13)	− 0.08 (− 0.36, 0.20)	−0.13 (− 0.35, 0.09)	0.96
Waist Circumference (cm)				
Prenatal	− 1.3 (− 3.3, 0.6)	− 0.6 (− 3.0, 1.8)	− 1.7 (− 4.2, 0.7)	0.62
Average 1–5 years	0.4 (− 2.4, 3.3)	0.2 (− 4.0, 4.3)	− 1.6 (− 4.9, 1.7)	0.51
8 Years	− 0.5 (− 2.1, 1.2)	− 1.0 (− 3.1, 1.1)	−1.3 (− 3.3, 0.6)	0.71
Body Fat %				
Prenatal	− 0.8 (− 2.2, 0.6)	− 0.6 (− 2.1, 0.9)	−0.6 (− 2.6, 1.3)	0.79
Average 1–5 years	0.4 (− 1.7, 2.4)	− 0.4 (− 3.0, 2.1)	−1.1 (− 3.8, 1.6)	0.82
8 Years	0.0 (− 1.2, 1.2)	−0.8 (− 2.1, 0.5)	−0.4 (− 2.0, 1.2)	0.73

^a^All estimates are adjusted for maternal race, education, marital status, age at delivery, income, prenatal vitamin use, delivery method, maternal BMI, and prenatal serum cotinine concentrations. Both waist circumference and body fat percentage are also adjusted for child’s age. All urinary triclosan concentrations have been creatinine standardized

^b^For prenatal analysis All: *N* = 220, Boys: *N* = 99, Girls: *N* = 121. For early childhood analysis All: *N* = 212, Boys: *N* = 94, Girls: *N* = 118. For age 8 year analysis All: *N* = 218, Boys: *N* = 99, Girls: *N* = 119

^c^Effect measure modification *p*-values were determined using a two sample z-test to compare the effect estimates in boys and girls

**Table 3:** Adjusted difference in adiposity measures at age 8 years per 10-fold-increase in urinary triclosan concentrations among HOME Study children: Adjusted for all triclosan measures^a, b,c,d^

Adiposity Outcome and Triclosan Measure Timing^a^	All, Adjusted Difference (95% CI)	Boys, Adjusted Difference ^c^ (95% CI)	Girls, Adjusted Difference ^c^ (95% CI)	EMM *p*-value^d^
BMI z-score				
Prenatal	−0.06 (− 0.28, 0.16)	0.05 (− 0.30, 0.39)	−0.13 (− 0.42, 0.15)	0.43
Early Childhood	0.11 (−0.24, 0.46)	0.11 (− 0.50, 0.72)	−0.05 (− 0.42, 0.15)	0.57
8 Years	− 0.07 (− 0.27, 0.12)	−0.14 (− 0.46, 0.17)	−0.10 (− 0.34, 0.14)	0.90
Waist Circumference				
Prenatal	− 1.6 (− 3.6, 0.4)	− 0.8 (− 3.4, 1.7)	−1.7 (− 4.2, 0.7)	0.67
Early Childhood	1.3 (−1.8, 4.3)	1.8 (− 2.8, 6.4)	−0.9 (− 4.4, 2.6)	0.35
8 Years	− 0.5 (− 2.3, 1.2)	− 1.5 (− 3.9, 0.8)	−0.9 (− 3.0, 1.2)	0.43
Body Fat %				
Prenatal	−0.9 (− 2.4, 0.5)	− 0.5 (− 2.1, 1.0)	−0.7 (− 2.7, 1.3)	0.84
Early Childhood	0.6 (− 1.7, 2.8)	0.6 (−2.2, 3.4)	−1.0 (− 3.8, 1.8)	0.70
8 Years	0.1 (− 1.2, 1.4)	−0.9 (− 2.3, 0.6)	− 0.1 (− 1.9, 1.6)	0.52

^a^Along with mutually adjusting for all three triclosan measures, all estimates are adjusted for maternal race, education, marital status, age at delivery, income, prenatal vitamin use, delivery method, maternal BMI, and prenatal serum cotinine concentrations. Both waist circumference and body fat percentage have also been adjusted for child’s age. All urinary triclosan concentrations have been creatinine standardized

^b^For all analysis All: *N* = 210, Boys: *N* = 94, Girls: *N* = 116

^c^Effect estimates and 95% CI were derived from a sex-stratified model

^d^Effect measure modification *p*-values were determined using a two sample z-test to compare the effect estimates for boys and girls

Among the boys, the associations between urinary triclosan concentrations and measures of body composition were imprecise and inconsistent. After mutually adjusting for all three triclosan measures, the associations remained imprecise and inconsistent (Table 3). Notably, the association between early child triclosan concentrations and waist circumference went from being weakly inverse before adjustment for triclosan concentrations in other periods to modestly positive after adjustment (Table 3).

### Secondary analysis

Excluding children whose mothers had gestational diabetes or a hypertensive disorder during pregnancy, including breastfeeding as a covariate, including diet and physical activity as covariates, or excluding children with birth weight z-scores ≤10th percentile for gestational age did not appreciably change the association of prenatal, early childhood, and 8-year triclosan concentrations with child adiposity (Additional file 1: Table S2). Our results for prenatal triclosan were not meaningful different when we adjusted for urinary BPA, ∑DEHP, MBzP, MEP, MCPP, MnBP, MiBP, PFOA, and PBDE-47 (Additional file 1: Table S3).Using different statistical approaches to account for urine dilution produced estimates similar to those presented in Table 2 for all three adiposity measures (Additional file 1: Table S4).

## Discussion

In this cohort, urinary triclosan concentrations during pregnancy, early childhood, and at 8 years of age were not associated with measures of childhood adiposity at age 8 years. We observed some evidence that prenatal urinary triclosan concentrations were inversely and weakly associated with some adiposity measures among girls, but these associations were not significantly different from those in boys.

Consistent with our work, a prior prospective study reported a null association between maternal urinary triclosan concentrations during pregnancy and child adiposity at age 4 to 9 years and no sex-specific patterns were observed [22]. Buckley et al. used a single urine sample from women in the third trimester, whereas we quantified urinary triclosan concentrations at 16 and 26 weeks of gestation. Another prospective study found weak positive associations between 2nd trimester urinary triclosan concentrations and weight among boys at 1, 2, and 3 years of age; however, the 95% confidence intervals for these associations included the null [23]. This prior study did not examine adiposity in girls. In the present study, the estimates among boys were imprecise and close to the null; however, our study may have not had sufficient statistical power to detect associations in boys.

Several investigators have reported varying results when examining the cross-sectional association between urinary triclosan and body composition. A study using NHANES data (2007–2010) examining children and adolescents reported no association of urinary triclosan concentrations with body weight, BMI z-score, and waist circumference [17]. Another study using NHANES data from 2003 through 2010, which included children and adults, found that urinary triclosan concentrations were associated with decreased BMI and waist circumference among both children and adults [19]. In contrast, Lankester et al. found higher BMI among adult NHANES participants with detectable urinary triclosan concentrations compared to individuals with undetectable concentrations of triclosan [21]. It is not apparent why different studies using overlapping sets of NHANES data have produced such discrepant results. In a cross-sectional study conducted among 76 Indian children, there was no significant association between urinary triclosan concentrations and adiposity, and average triclosan concentrations were slightly lower for obese/overweight as compared to non-obese children, a result which was similar to a study among obese and overweight Belgian adults [18, 20].

A strength of our study is that mothers and children were followed prospectively starting at 16 weeks of gestation until children were 8 years old. This allowed us to assess triclosan concentrations during several distinct developmental periods and identify potentially sensitive periods of development to triclosan exposure.

A potential reason for discrepancies across this and prior studies could be misclassification of triclosan exposure. Almost all previous studies have relied on one urine sample to assess triclosan exposure. Because triclosan has a biological half-life of ~ 21 h, does not persist in the human body, and exposures are likely episodic in nature, there is the potential for exposure misclassification, which would reduce statistical power for detecting potential associations and attenuate effect estimates towards the null if exposure misclassification is non-differential with respect to child adiposity [41, 42]. We collected urine samples twice during pregnancy and annually from 1 to 5 years of age, allowing us to reduce within-person variability by averaging triclosan concentrations from multiple measures during these periods. It should be noted that we averaged annual triclosan concentrations between ages 1 to 5 years to represent early childhood exposure; not all children provided a sample every year. The missing urine samples for some children between the ages 1 to 5 years may have led to misclassification of the early childhood triclosan exposure. This, along with small sample size in sex-stratified analyses, might explain the imprecise effect estimates among both sexes for this time period.

By averaging urinary triclosan concentrations during the prenatal and early childhood periods we were not able to identify finer windows of vulnerability within these periods. However, given the potential for exposure misclassification during early childhood, as demonstrated by the poor reproducibility of repeated childhood triclosan measures, averaging individual measurements will reduce exposure misclassification. Sampling conditions, such as the time of day, can also impact the variability of urinary biomarkers, including triclosan [43]. We were unable to include time of collection in our analysis as these data were unavailable, and this may have resulted in exposure misclassification. However, previous studies have shown that adjusting for sample collection conditions did not result in substantially different ICCs compared to the ICCs calculated without such adjustments [44]. Furthermore, non-detectable urinary triclosan concentrations can be problematic for statistical analyses (% of values <LOD ranged from 5.3% at the 8-year visit to 34% at 1-year visit), prior work shows that when ≤ 25% of values are <LOD, substitution of LOD/√2 provides results similar to other methods used to treat left-censored exposures [45].

A strength of our study is that we used three different measures of child adiposity that are all highly correlated with dual X-ray absorptiometry derived measures of total and trunk fat mass [28]. However, there remains the potential for misclassification when using BMI as a measure of adiposity, because it reflects both fat and lean mass and can potentially underestimate body fat mass [28, 46]. Other measures of adiposity, such as densitometry and dual energy X-ray absorptiometry, can quantify fat and lean mass to provide a more accurate phenotype of adipose tissue mass and distribution. Future research could examine the relationship between urinary triclosan concentrations and more precise body composition measures in children.

Another strength of this study was the ability to control for numerous socioeconomic, perinatal, and environmental factors. Specifically, we were able to control for many early life factors associated with later life adiposity, including delivery method, maternal BMI, prenatal tobacco smoke exposure, and breastfeeding. In addition, our results were similar after a variety of adjustments, including prenatal exposure to other environmental chemicals. However, we were only able to control for relatively crude measures of diet and physical activity at age 8 years. To better control for diet and physical activity, future studies could use 24-h food recalls as a more comprehensive measure of children’s diets and activity monitors can more accurately assess activity among children [47]. In addition, we did not evaluate how potentially obesogenic chemicals could act together to impact body composition and this remains an important avenue for future research.

We hypothesized that early life triclosan exposure could affect child body composition by disrupting thyroid hormone homeostasis or the gut microbiome. In rodents, triclosan exposure causes decreased circulating thyroxine concentrations and there is suggestive evidence of an association in some, but not all, epidemiological studies [48–51]. Prior studies in rodents and fish observed that triclosan exposure altered some aspects of the gut microbiome [10, 11]. Results in humans are inconclusive; a small crossover study of 16 healthy adults did not observe changes in gut microbiome diversity after using triclosan containing products [12], but another study of children observed that triclosan was associated with some features of the gut microbiome [52]. Given the potential importance of thyroid hormones and the gut microbiome in the etiology of other childhood health outcomes [53–55], future research would benefit from studying the relationships of triclosan exposure with these biological mechanisms and related health outcomes.

## Conclusion

In this cohort, we did not find compelling evidence of an association between early life triclosan exposure and childhood adiposity at age 8 years. Given the potential for triclosan exposure to affect thyroid hormone homeostasis and the microbiome, future studies could examine these and related health endpoints.

## Additional file

Additional file 1: Results of all secondary analysis and DAGs. (ZIP 160 kb)

## Abbreviations

BDE-47: Brominated diphenyl ether-47
BMI: Body mass index
BPA: Bisphenol A
CCHMC: Cincinnati Children’s Hospital Medical Center
CDC: Centers for Disease Control and Prevention
DAG: Directed acyclic graph
DEHP: Di(2-ethylhexyl) phthalate
HOME: Health Outcomes and Measures of the Environment
ICC: Intraclass correlation coefficient
IRB: The institutional review boards
LOD: Limit of detection
MBzP: Monobenzyl phthalate
MCPP: Mono(2-carboxypropyl) phthalate
MEP: Monoethyl phthalate
MiBP: Mono-iso-butyl phthalate
MnBP: Mono-n-butyl phthalate
NHANES: U.S. National Health and Nutrition Examination Survey
PFOA: Perfluorooctanoic acid
