# Supplementary material for: Early life Triclosan exposure and child adiposity at 8 Years of age: a prospective cohort study
Source: Environ Health. 2018 Mar 5;17:24. doi: 10.1186/s12940-018-0366-1 (PMC5838861; doi:10.1186/s12940-018-0366-1)
Supplement: Supplementary file 1 — Results of all secondary analysis and DAGs. (ZIP 160 kb) [file 12940_2018_366_MOESM1_ESM.zip › Additional File 1_KallooR2.docx]

**Additional Table 1:** Number of children with 1-5 urine samples between 1 and 5 years of age for the early childhood urinary triclosan exposure variable.

| **Number of Urine Samples** | **N of children** |
| --- | --- |
| **1** | 14 |
| **2** | 24 |
| **3** | 33 |
| **4** | 58 |
| **5** | 83 |

**Additional Table 2:** Adjusted difference in 8 year old BMI z-score per 10-fold increase in maternal, early childhood and 8 year urinary triclosan concentrations among HOME Study women and their children. ^a, b^

| **Excluding Mothers With Gestational Diabetes** | **Boys, Adjusted Difference**  **(95% CI)** | | **Girls, Adjusted Difference**  **(95% CI)** | |
| --- | --- | --- | --- | --- |
| TCS-Average Pre Natal (n= 210) | 0.06 | (-0.26, 0.37) | -0.17 | (-0.46, 0.12) |
| TCS- Average 1-5 years (n= 202) | 0.00 | (-0.54, 0.55) | -0.11 | (-0.49, 0.26) |
| TCS- 8 Years (n= 210) | 0.01 | (-0.27, 0.29) | -0.18 | (-0.39, 0.04) |
|  |  |  |  |  |
| **Excluding Mothers With Hypertensive Disorders During Pregnancy** | **Boys, Adjusted Difference**  **(95% CI)** | | **Girls, Adjusted Difference**  **(95% CI)** | |
| TCS-Average Pre Natal (n= 219) | 0.05 | (-0.26, 0.36) | -0.18 | (-0.47, 0.09) |
| TCS- Average 1-5 years (n= 211) | 0.14 | (-0.39, 0.68) | -0.17 | (-0.54, 0.20) |
| TCS- 8 Years (n= 217) | -0.04 | (-0.32, 0.24) | -0.16 | (-0.37, 0.05) |
|  |  |  |  |  |
| **Including Diet and Physical Activity** | **Boys, Adjusted Difference**  **(95% CI)** | | **Girls, Adjusted Difference**  **(95% CI)** | |
| TCS-Average Pre Natal (n= 220) | -0.00 | (-0.43, 0.43) | 0.11 | (-0.27, 0.50) |
| TCS- Average 1-5 years (n= 212) | 0.42 | (-0.52, 1.35) | -0.30 | (-0.79, 0.18) |
| TCS- 8 Years (n= 218) | -0.04 | (-0.21. 0.36) | -0.23 | (-0.51, 0.04) |
|  |  |  |  |  |
| **Including Breastfeeding Variable** | **Boys, Adjusted Difference**  **(95% CI)** | | **Girls, Adjusted Difference**  **(95% CI)** | |
| TCS-Average Pre Natal (n= 220) | 0.14 | (-0.59, 0.30) | -0.09 | (-0.37, 0.20) |
| TCS- Average 1-5 years (n= 212) | -0.01 | (-0.56, 0.53) | -0.12 | (-0.49, 0.24) |
| TCS- 8 Years (n= 218) | -0.07 | (-0.35, 0.22) | -0.14 | (-0.37, 0.08) |
| **Excluding Children Below 10^th^ Percentile of Birth Weight z-score For Gestational Age** | **Boys, Adjusted Difference**  **(95% CI)** | | **Girls, Adjusted Difference**  **(95% CI)** | |
| TCS-Average Pre Natal (n=197) | 0.21 | (-0.10, 0.52) | -0.18 | (-0.47, 0.09) |
| TCS- Average 1-5 years (n=190) | 0.17 | (-0.36, 0.71) | -0.13 | (-0.50, 0.25) |
| TCS- 8 Years (n=195) | -0.10 | (-0.38. 0.17) | -0.15 | (-0.38, 0.08) |

a-All estimates are adjusted for maternal race, education, marital status, age at delivery, income, prenatal vitamin use, delivery method, maternal BMI, and prenatal cotinine levels.
b-Body fat percentage and waist circumference had similar results (BMI z-score and waist circumference r=0.80, BMI z-score and body fat percentage r=0.84)

**Additional Table 3:** Sex stratified adjusted difference in 8 year old waist circumference per 10-fold increase in maternal urinary triclosan concentrations among HOME Study women and children, each row represents a separate model which included the potentially obesogenic chemical listed. ^a^

| **Chemical** | **Pearson's Correlation Coefficient** | **Waist Circumference  Prenatal β (CI):** | |
| --- | --- | --- | --- |
|  |  | **Boys** | **Girls** |
| Adjusted Model |  | -0.6 (-3.0, 1.8) | -1.7 (-4.2, 0.7) |
| BPA | 0.14 | -0.5 (-1.4, 3.7) | -1.6 (-4.1 0.9) |
| DEHP | 0.05 | -0.5 (-2.9, 1.9) | -1.7 (-4.2, 0.8) |
| MBzP | -0.02 | -0.7 (-3.1, 1.7) | -1.7 (-4.1, 0.8) |
| MEP | 0.04 | -0.5 (-3.0, 1.8) | -1.6 (-4.1, 0.8) |
| MCPP | 0.06 | -0.6 (-3.0, 1.8) | -1.5 (-4.0, 0.9) |
| MnBP | 0.11 | -0.7 (-3.2, 1.7) | -1.1 (-3.5, 1.4) |
| MiBP | -0.10 | -0.6 (-3.0, 1.9) | -1.6 (-4.0, 0.8) |
| PFOA | -0.04 | -1.0 (-3.4, 1.4) | -0.9 (-3.2, 1.5) |
| PBDE-47 | -0.04 | 0.2 (-2.0, 2.4) | -1.1 (-3.4, 1.2) |

a-All estimates are adjusted for maternal race, education, marital status, age at delivery, income, prenatal vitamin use, delivery method, maternal BMI, and prenatal cotinine levels.

**Additional Table 4.** Adjusted difference in BMI z-score per 10-fold increase in prenatal, early childhood, and 8 year urinary triclosan concentrations among women and children in the HOME Study using different methods of adjusting for urine dilution. ^a b^

| **Non-Creatinine Adjusted** | **Boys, Adjusted Difference**  **(95% CI)** | | **Girls, Adjusted Difference**  **(95% CI)** | |
| --- | --- | --- | --- | --- |
|  |  | |  | |
| TCS-Average Pre Natal | 0.11 | (-0.19, 0.40) | -0.24 | (-0.52, 0.03) |
| TCS- Average 1-5 years | 0.42 | (-0.06, 0.90) | -0.08 | (-0.44, 0.29) |
| TCS- 8 Years | -0.06 | (-0.32, 0.20) | -0.03 | (-0.31, 0.25) |
| **Creatinine as Covariate** | **Boys, Adjusted Difference**  **(95% CI)** | | **Girls, Adjusted Difference**  **(95% CI)** | |
| TCS-Average Pre Natal | 0.06 | (-0.25, 0.37) | -0.22 | (-0.50, 0.05) |
| TCS- Average 1-5 years | 0.24 | (-0.27, 0.75) | -0.14 | (-0.52, 0.24) |
| TCS- 8 Years | -0.03 | (-0.31, 0.25) | -0.17 | (-0.38, 0.05) |
| **Creatinine Adjusted and as Covariate** | **Boys, Adjusted Difference**  **(95% CI)** | | **Girls, Adjusted Difference**  **(95% CI)** | |
| TCS-Average Pre Natal | 0.06 | (-0.25, 0.37) | -0.22 | (-0.50, 0.06) |
| TCS- Average 1-5 years | 0.20 | (-0.31, 0.72) | -0.12 | (-0.50, 0.25) |
| TCS- 8 Years | -0.03 | (-0.31, 0.25) | -0.17 | (-0.38, 0.05) |

a-All estimates are adjusted for maternal race, education, marital status, age at delivery, income, prenatal vitamin use, delivery method, and prenatal cotinine levels.

Body fat percentage and waist circumference had similar results (BMI z-score and waist circumference r=0.80, BMI z-score and body fat percentage r=0.84)

b- For the prenatal analysis All: N=220, Boys: N=99, Girls: N=121. For the early childhood analysis All: N=212, Boys: N=94, Girls: N=118. For the age 8 years analysis All: N=218, Boys: N=99, Girls: N=119.

**Additional Figure 1**: A directed acyclic graph of the relationship between maternal urinary triclosan concentrations and adiposity risk in the HOME Study.

**Additional Figure 2**: A directed acyclic graph of the relationship between childhood urinary triclosan concentrations and adiposity risk in the HOME Study.
