# Supplementary figures and images for: Early life Triclosan exposure and child adiposity at 8 Years of age: a prospective cohort study
Source: Environ Health. 2018 Mar 5;17:24. doi: 10.1186/s12940-018-0366-1 (PMC5838861; doi:10.1186/s12940-018-0366-1)

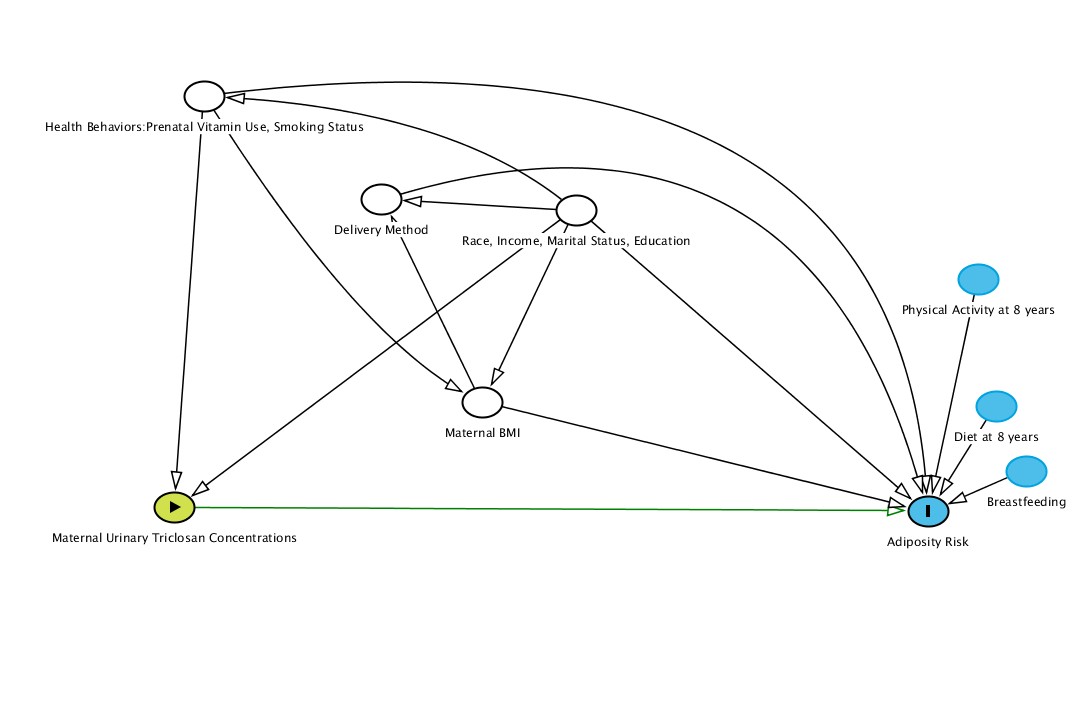

Supplement: Supplementary file 1 — Results of all secondary analysis and DAGs. (ZIP 160 kb) [file 12940_2018_366_MOESM1_ESM.zip › Additional Figure 1_KallooR2.jpg]

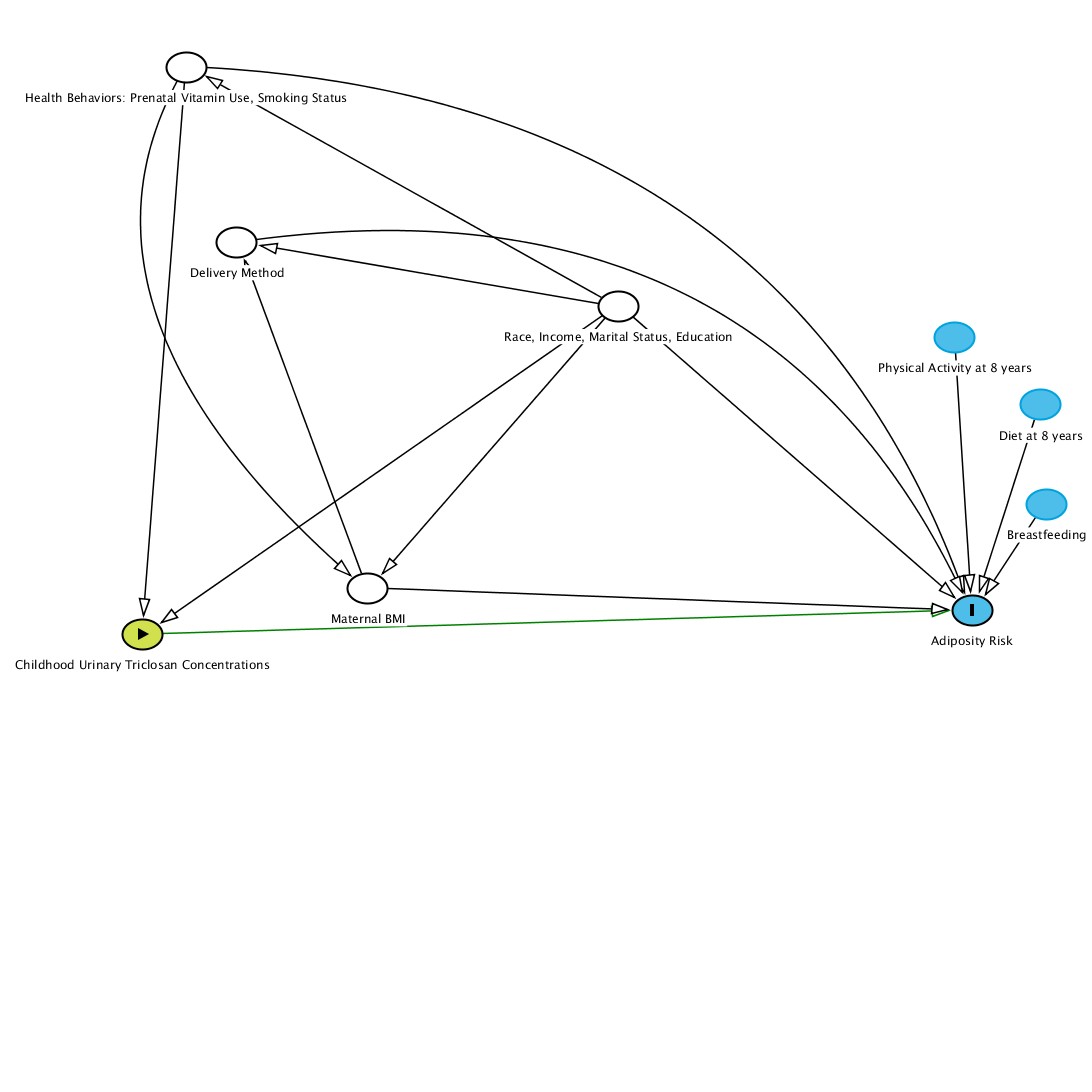

Supplement: Supplementary file 1 — Results of all secondary analysis and DAGs. (ZIP 160 kb) [file 12940_2018_366_MOESM1_ESM.zip › Additional Figure 2_KallooR2.jpg]
